# A response for substance and harm reduction in Pacific Island countries and territories

**DOI:** 10.1186/s12954-015-0080-z

**Published:** 2015-10-16

**Authors:** Robert Power, Lucina Schmich, Vili Nosa

**Affiliations:** Centre for International Health, Burnet Institute, 85 Commercial Road, GPO Box 2284, Melbourne, Victoria 3004 Australia; Allen + Clarke, (Wellington 6143), Level 2, The Woolstore, 262 Thorndon Quay, PO Box 10 730, Wellington, 6011 New Zealand; Pacific Health, School of Population Health, Faculty of Medical & Health Sciences, University of Auckland, Private Bag 92019, Auckland, New Zealand

**Keywords:** Alcohol policies, Alcohol, Drugs, Pacific populations, Pacific Island countries and territories

## Abstract

The Pacific is characterised as a region for the purposes of many international interventions and assistance programmes. Representatives of Pacific States participate in regional fora to build a strategic and unified approach to development. Regionally, bilateral trade agreements impact upon strategies to regulate alcohol imports. Policing and customs initiatives are increasingly supporting prevention of illicit drug production and trafficking, and model laws have been proposed to achieve consistency in enforcement. The aim of this commentary is to provide a response for policies using the case of alcohol and other drug research in the Pacific Islands Countries Territories. This commentary undertook a review of the current literature for regional developments for alcohol and other drug use in the Pacific Island Countries Territories region. A total of 14 articles were used in this article. The publication date for the articles used in this review ranged from 1997 to 2011. The findings of the review found that there should be a co-ordinated approach for adopting alcohol and other drug approaches. Furthermore, there should be a co-ordinated regional response with the inclusion of targeted domestic programming that will meet the needs for the Pacific Island countries and territories. Countries in the Pacific Island territories are characterised by varying degrees of political stability. Without stable government and democratic process, it is likely to remain difficult to develop consistent and effective legislation and policy for implementation of successful alcohol and other drug programmes. We found that there is a lack of robust and current data for alcohol and other drugs in Pacific Island countries and territories. Further research funding is needed to build the limited knowledge of alcohol and other drug substance use.

## Commentary

The Pacific region has a number of other social issues. Many Pacific Island countries and territories (PICTS) are dependent on aid to support their countries’ infrastructures. In addition, many PICTs are isolated in the region, which can hinder access to healthcare and other drug and alcohol harm reduction interventions. Indeed, many PICTS have no interventions specifically designed for their own situation and culture, and many of these countries send patients to New Zealand or Australia. There is also an increase of migration to larger metropolitan countries such as New Zealand and Australia, which can have a negative impact on a community’s human resource capacity.

The PICTs comprise people of Melanesian, Micronesian, and Polynesian background. Countries such as Fiji and the Solomon Islands include large, later-generation populations of Chinese and Indians. Many of the PICTs are characterised by high rates of unemployment, rural urban drift, and high youth populations (in some cases over 50 percent under 20 years). These factors contribute to significant regional-level population mobility. Registered seasonal employer programmes (RSE) and other programmes provide job opportunities for skilled and unskilled workers [1]. Workforces range from Fiji as an exporter of skilled labour, to large numbers of seafarers from Tuvalu and Kiribati working abroad, to the new recruits in the seasonal migrant labour schemes to Australia and New Zealand.

The purpose of this paper is to provide a response for policies using the case of alcohol and other drug research in the Pacific Island countries and territories (PICT).

### Research methods

This study carried a literature review for Pacific regional developments plus alcohol and other drug use in the Pacific region. A comprehensive literature search was conducted on various databases. These included, Google Scholar, Scopus, PubMed, Embase, PsycINFO and Medline. Key terms such as Pacific Island countries and territories, Pacific regional developments, Pacific alcohol and other drugs research, alcohol in the Pacific, substance use in the Pacific, drugs and alcohol, Pacific Islands substance, Pacific alcohol and drug use in NZ, Pacific alcohol and drug use in Australia, Pacific alcohol and drug use in the USA were entered into these databases, and a total of 50 articles were extracted. The scope of the documents and literature were accessed through various websites. A total of 14 articles were extracted and included in this review. Relevant articles were assessed and selected based on the inclusion/exclusion of articles written in English and the quality and validity of the data that fitted the research criteria. The findings from each article were tabled and examined using a thematic analysis for common similarities.

### Key findings

#### Substance use in the pacific region

Studies of Pacific Islanders born abroad, including New Zealand, Australia, and the USA provide some insights into the impact of changing economic circumstances and mobility. Existing research highlights unique context-driven risk and protective factors beneficial for illustrating uniquely Pacific issues. The study by Schmich & Power [2] flagged community policing programmes, peace keeping, industry-specific migrant labour, and the return of “troubled” youth to the care of extended families in the islands as issues of concern relevant to alcohol and other drug (AOD) use. Significantly, Khan et al. [3] identified that amongst Pacific Islanders living in Australia, parental disciplinary methods impacted heavily upon the likelihood of youth involvement in problematic behaviours, particularly substance use. Due to high youth populations in the PICTs, much of the existing data and programming is targeted towards this group. The role of parental substance use and responses to youth substance in the PICTs may be similarly influential, and yet adult behaviours and their role in the response remain largely unexplored.

In many PICTs where cannabis is an issue, the economic imperatives borne out of limited income-earning opportunities have been cited as a reason for increased production, and anecdotal evidence suggests that the growing and manufacturing of cannabis is a cash crop for many families in Fiji and the Cook Islands. Structures and functions often mirror “legitimate” business, with economic models existing inside the illegal milieu [4]. The same imperative applies to cultivation, production, and sale of other harmful, but not illegal drugs, such as alcohol, tobacco, and kava. In Papua New Guinea in the late 1980s, private interests declared that the economic loss would prove disastrous if tobacco plants were to close down [5]. The same types of arguments are posited in response to increased regulation of the alcohol industry.

In 2004/05 a situational assessment of illicit drug use in the Asia Pacific Region reported a dearth of data in the Pacific. In an endeavour to fill this gap, in 2008, the Australian National Council on Drugs (ANCD) commissioned a further situational assessment of drug-use issues for 16 Pacific Island countries and territories (PICTs). All the countries selected for the analysis were recipients of AusAID development grants: Cook Islands, Federated States of Micronesia, Fiji, Kiribati, Marshall Islands, Nauru, Niue, Palau, Papua New Guinea (PNG), Samoa, Solomon Islands, Timor Leste, Tonga, Tuvalu, and Vanuatu [2].

A total of 15 countries that were part of this study data were collected from key informants working in the Ministry of Health who are responsible for alcohol and other drug-use statistics. This was supplemented with an extensive review of published materials and grey literature. Only a limited number of peer-reviewed publications were available, and the analysis was largely informed by project and government reports, census data, and household expenditure surveys and a review of websites, media, and anecdotes.

Emerging issues include reports of increasing levels of cannabis, kava, alcohol and inhalant use, and correlations between drug use and violence. Information about use of amphetamine-type substances and other stimulants is equivocal, but reports are increasing. Recent amphetamine-type stimulant (ATS) seizures in the region suggest there is cause for concern [5]. Regional bodies such as the Oceania Customs Organisation (OCO) have identified geographical and structural risk factors that make the Pacific an ideal transhipment point for illegal drugs, including sparse populations, remoteness, and isolation. Key informants reported limited flow of information between agencies across all sectors as a barrier to any concerted response. The situational analysis report details country by country results [6]. In this article, we extrapolate key findings and issues of broad regional significance.

Kava is also another key substance which plays an important part in Pacific life through ceremony and ritual. It has also become an important source of export income for the region. At its peak, the industry was estimated to be worth US$200 million. However, international bans were introduced due to liver toxicity associated with dietary supplements containing kava extract. A subsequent WHO research report has linked this to chemicals used in the extraction process and not kava itself [7]. After 6 years of international bans, the International Kava Executive Council expects the kava trade to return to normal with great potential to reinvigorate the export industry and thus local economies and employment opportunities. From a health perspective, key informants interviews from Schmich & Power [2] study reported both positive and negative impacts associated with kava use. These include social, familial, and financial burdens associated with regular and excess kava consumption. By way of contrast, some informants and reports suggested that kava is a less harmful alternative to alcohol which is associated with violence. There is still scope to explore some changing patterns of consumption, including the shift from ritual and ceremonial consumption to the emergence of kava bars, and anecdotal reports of increased youth consumption particularly amongst young women. Other anecdotal reports suggest that amongst the Fijian and Tongan communities in New Zealand, a relationship exists between the consumption of kava at “kava clubs” and a “washdown” process where alcohol is consumed to encourage intoxication. Thus, there is a need to explore the context, patterns, and consequences of consumption.

Schmich & Power [2] also identified alcohol—licit and illicit (toddy and homebrew)—and cannabis as the substances of greatest concern. These findings are based on youth risk behaviour surveys in Vanuatu, Tonga, and Micronesia; media reports; and key informant questionnaires and interviews.

The importation of alcohol and tobacco continues to be a problem for many PICTS. Furthermore, heavy alcohol use continues to be a problem, and a number of alcohol-related problems such as drinking and driving, physical violence, mental health, and heavy binge drinking continue to be major issues in the region [6, 8–11].

The analysis also outlines a number of contextual factors influencing drug-use patterns and trends in the Pacific, including the following: governmental stability, exposure to foreign visitors, migration patterns, culture, and religion. It also identifies varying degrees of political stability across a number of counties and the difficulty for developing effective legislation and policy to facilitate the implementation of AOD programmes.

### Data gaps for alcohol and other drugs

There is still limited data available on licit and illicit AOD use in the Pacific, but that which exists identifies a number of patterns, trends, and areas for further research and intervention. Significantly, and in contrast to Asia, injecting drug use is not common. A 2008 review conducted to assist development of the UNAIDS Commission on AIDS in the Pacific [9] found that injecting most often occurs in the American-affiliated states, such as French Polynesia and Palau, but little if any occurs in other PICTs. Perhaps due to the relative lack of evidence for injecting drug use in the region, the Pacific Regional Strategy on HIV/AIDS 2004-2008 [9] identified sex work and high rates of sexually transmitted infections as key risk factors, whilst the contribution of substance use as a risk was largely overlooked.

Migrants returning from abroad have been identified as bringing new attitudes and lifestyles—including AOD use—into a setting where AOD service provision is in its infancy. Where AOD services exist, they are often located within mental health service systems and have the capacity to cater only for complex care patients. No large-scale evaluations of this service model in the Pacific context have been identified. However, a 2009 qualitative study in the Solomon Islands noted that traditional community-response mechanisms struggled to manage substance use in mental health services [12–14].

Importantly, the segregation or lack of coordination across services often impacts on the collection, recording, and reporting of information. For example, some HIV programmes include youth-focussed activities around AOD use, but it remains difficult to capture the effectiveness of such activities. No local organisations mandated to address both licit and illicit AOD issues and advocate for change were identified during the ANCD study.

### Harm minimisation for AOD policy approaches

The concept of harm minimisation appears relatively new for policy makers in the region, and this poses a further challenge for developing responses. Furthermore, lack of support for developing national responses to problematic alcohol use and the absence of legislative frameworks for responding were reported as barriers. In addition, when weighed against other health issues, addressing substance-use issues is not always considered an urgent health priority.

With respect to legislation, since the 1992 Honiara Declaration on Law Enforcement Cooperation, the Pacific Island Forum Secretariat (PIFS) has been instrumental in coordinating the response to international crime in the region. In 2005, a PIFS representative noted that there is no regional- or country-based policy for AOD issues. A number of countries have announced law reforms and reviews of existing illicit drugs legislation, yet the shape of these reforms remains unknown. A joint working group including members from regional law enforcement agencies is also designated to address drug control issues, and the 2002 PIFS-sponsored Illicit Drugs Control Bill was to form the basis of new legislation for narcotics control to be applied across the region [8]. It was used as the basis for legislation in Tonga with a modified version to be considered in the PNG Parliament—the outcome of both is not yet known. Internationally, the most commonly used mechanisms for regulating alcohol consumption include taxes, licences, and bans. Whilst a number of these are employed across the Pacific, they fail to account for unregulated and illegally produced toddy and homebrew. Key informants in the ANCD research noted that standard regulatory measures tend to increase homebrew production. In addition, where significant important revenue is generated from direct sales, licencing, and taxation, the motivation to regulate and reduce consumption may be limited.

### Conclusion and recommendations

International development programming in the Pacific provides the foundation for framing a response to AOD use, with successive programmes highlighting risks and challenges for working in the Pacific. An effective response depends on using existing programmes and data sources, whilst recognising that the evidence-base for effective interventions needs to be improved. In addition, engaging existing service providers and recognising the cultural and economic context are essential for success.

Schmich & Power’s [2] study recommends focussing on building up the capacity of existing data collection systems to amass regular data on AOD use trends, and advocate for inclusion of specific AOD questions in routine surveys. There is also an opportunity to collaborate with regional initiatives such as the UNODC Global Smart Program whereby initiatives for the government to collect and report data on illicit drug use. This would be a good starting point to monitor illicit drug use.

As key members of the secretariat for the Pacific Drug and Alcohol Research Network which was established in 2005 in response to a lack of data describing drug and alcohol issues in Pacific Island countries and territories, the group has met regularly since that time bringing together ministry of health, non-government, multi-lateral, and law enforcement representatives with university researchers and public health workers to gather and report current data, undertake capacity building activities, and create opportunities to collaborate on research projects and program implementation activities.

Operational and action research needs to be encouraged and fully funded. There is a need to develop and promote research programmes to understand and respond to trends and impacts as they emerge, and enhance local evidence bases. A key focus should investigate the extent of social, economic, and health consequences of AOD use in Pacific Island countries and territories. Subsequent findings should be used to develop appropriate responses in the light of evidence-informed interventions successfully adopted in other contexts, including those within the paradigm of harm reduction.
